# Anxiolytic and cognitive effects of dietary phenolics in zebrafish: rosmarinic acid, gallic acid, and 3-hydroxytyrosol

**DOI:** 10.3389/fphar.2026.1801073

**Published:** 2026-04-29

**Authors:** Tugba Ucar Akyurek, Ilkay Erdogan Orhan, Fatma Sezer Senol Deniz, Ion Brinza, Razvan Stefan Boiangiu, Lucian Hritcu

**Affiliations:** 1 Department of Pharmacognosy, Faculty of Pharmacy, Gazi University, Ankara, Türkiye; 2 Department of Pharmacognosy, Faculty of Pharmacy, Lokman Hekim University, Ankara, Türkiye; 3 Faculty of Sciences, Lucian Blaga University of Sibiu, Sibiu, Romania; 4 Department of Biology, Faculty of Biology, Alexandru Ioan Cuza University of Iasi, Iasi, Romania

**Keywords:** 3-hydroxytyrosol, anxiety, gallic acid, memory, rosmarinic acid, zebrafish

## Abstract

**Introduction:**

Rosmarinic acid (RA), gallic acid (GA), and 3-hydroxytyrosol (3-HT) are phenolic compounds abundant in medicinal plants traditionally used to support cognitive function and neurological wellbeing. Although their antioxidant and neuroprotective properties are well documented in mammalian systems, their behavioral effects remain insufficiently explored in alternative *in vivo* models relevant for ethnopharmacological screening.

**Methods:**

In this study, we investigated the anxiolytic and cognitive-enhancing potential of RA, GA, and 3-HT in adult zebrafish (*Danio rerio*). Compounds were administered by immersion (3 μg/L, once daily for 8 days). Anxiety-related behaviors were evaluated using the novel tank diving test (NTT). At the same time, cognitive performance was assessed using the Y-maze and novel object recognition (NOR) tests to examine spatial and recognition memory, respectively.

**Results:**

RA significantly increased the top/bottom time ratio (*p* < 0.0001), indicating reduced anxiety_like behavior; however, this effect was accompanied by reductions in locomotor activity (distance traveled, *p* < 0.05; swimming velocity, *p* < 0.01), suggesting a possible sedative component. In contrast, GA and 3-HT elicited anxiety_like behavioral responses in the NTT without significantly affecting locomotion. Despite their anxiogenic profile, GA and 3-HT significantly enhanced spatial memory in the Y_maze test (*p* < 0.001), whereas RA and 3-HT increased exploration of the novel arm (*p* < 0.001). Moreover, 3-HT significantly improved recognition memory in the NOR test (*p* < 0.01).

**Discussion:**

Together, these findings reveal distinct neurobehavioral profiles for each phenolic compound: RA exhibited pronounced anxiolytic activity, while GA and 3-HT primarily enhanced cognitive performance, with 3-HT exerting broad-spectrum memory-facilitating effects. Overall, these results underscore the value of zebrafish as a translational model for behavioral pharmacology and support the ethnopharmacological relevance of RA, GA, and 3-HT as potential candidates for further investigation in the context of anxiety and cognitive disorders.

## Introduction

Neurodegenerative diseases are characterized by the progressive deterioration of neuronal structure and function and represent a growing global health burden with limited effective therapeutic options (Ding et al., 2022; Chen et al., 2024). Their multifactorial etiology, encompassing genetic susceptibility, environmental factors, protein misfolding, oxidative stress, and neuroinflammation, complicates both diagnosis and treatment strategies (Lin and Beal, 2006; Soto et al., 2018; Teleanu et al., 2022; Hong et al., 2025). These disorders are commonly classified based on their predominant clinical manifestations, affected brain regions, or underlying molecular pathology (Dugger and Dickson, 2017). A shared pathological hallmark across many neurodegenerative conditions is localized neuronal cell death, which is minimal in the healthy adult brain but markedly increased during disease progression (Chi et al., 2018). Despite extensive research efforts, drug development for neurodegenerative diseases remains challenging due to the complexity of the central nervous system, the restrictive blood-brain barrier, heterogeneous patient responses, and the typically slow and insidious nature of disease progression (Honig, 2015; Hang et al., 2023). Consequently, there is increasing interest in natural products, particularly plant-derived phenolic compounds, owing to their pleiotropic pharmacological properties, including antioxidant, anti-inflammatory, and neuroprotective effects (Angeloni and Vauzour, 2019; Cui et al., 2023). Among these, rosmarinic acid (RA), gallic acid (GA), and 3-hydroxytyrosol (3-HT) are widely distributed phenolics in medicinal plants traditionally used to support cognitive function and neurological health. Previous mammalian studies have demonstrated their neuroprotective potential in both *in vitro* and *in vivo* models. In our earlier work, these compounds were shown to inhibit β-site amyloid precursor protein cleaving enzyme 1 (BACE1), acetylcholinesterase (AChE), and butyrylcholinesterase (BChE), and to modulate Alzheimer’s disease (AD)-related gene expression, including *PSEN*, *APOE*, and *CLU* (Ucar Akyurek et al., 2024). *In silico* analyses further predicted favorable toxicity profiles and strong antioxidant capacity for all three phenolics (Ucar Akyurek et al., 2025). Despite this growing body of evidence, the neurobehavioral effects of RA, GA, and 3-HT have not been systematically evaluated in zebrafish (*Danio rerio*), a vertebrate model increasingly used in neuropharmacology due to its high genetic homology with humans, well-characterized behavioral repertoire, and suitability for high-throughput drug screening (McCutcheon et al., 2016; Sardela et al., 2018). Zebrafish offer several experimental advantages, including reproducible anxiety- and cognition-related behaviors, ease of compound administration, and strong translational relevance for early-stage behavioral pharmacology. Given the established neuroprotective potential of RA, GA, and 3-HT and the advantages of zebrafish as a model organism, the present study aimed to evaluate the anxiolytic and memory-enhancing effects of these phenolic compounds in adult *D. rerio*. To our knowledge, this is the first comprehensive behavioral assessment of RA, GA, and 3-HT in zebrafish using the novel tank diving test (NTT), Y-maze, and novel object recognition (NOR) test. The findings provide new insights into the distinct neurobehavioral profiles of these compounds and support their ethnopharmacological relevance as promising natural candidates for the management of anxiety and cognitive dysfunction associated with neurodegenerative disorders.

## Materials and methods

### Reagents

The phenolic compounds tested in this study with over 95% purity were acquired from Sigma-Aldrich (St. Louis, MO, United States).

### Animal care and study design

At the start of the study, 40 adult wild-type short-fin zebrafish (*D. rerio*), aged 5–7 months and measuring 3–4 cm in length, were obtained from the European Zebrafish Resource Center (Institute of Toxicology and Genetics, Germany). Fish of both sexes were used in equal proportions (1:1 male-to-female ratio). However, sex was not considered as a factor in the statistical analysis. All animals were acclimatized for at least 14 days under standard laboratory conditions before experimentation. During acclimatization, zebrafish were housed in groups of 10 per a 24 L thermostated tank maintained at 26 °C ± 1 °C, equipped with mechanical filtration and continuous aeration (dissolved oxygen: 7.20 mg/L), provided by Tetratec® air pumps (Tetra, Melle, Germany). A 14:10 h light/dark photoperiod was maintained, and fish were fed three times daily (08:00, 14:00, and 20:00) with Norwin Norvitall flakes (Norwin, Gadstrup, Denmark). Following acclimatization, fish were randomly assigned to four experimental groups (n = 10/group): control, rosmarinic acid (RA, 3 μg/L), 3-hydroxytyrosol (3-HT, 3 μg/L), and gallic acid (GA, 3 μg/L). Sample size determination was conducted using InVivoStat 4.7, an R-based statistical software package (Bate and Clark, 2014). Based on a significance level (p) of 0.05, a sample size of n = 10 zebrafish per group provided a statistical power of 96% to detect a biologically relevant effect size of 20%. This confirmed the adequacy of the group size for detecting meaningful behavioral differences. The concentration of 3 μg/L was selected based on previous zebrafish studies using immersion exposure, where low µg/L doses of bioactive compounds are sufficient to induce neurobehavioral effects due to efficient absorption through the gills and skin (Cachat et al., 2010; Sardela et al., 2018). This subtoxic concentration was chosen to allow detection of subtle behavioral changes without inducing locomotor impairment or nonspecific stress responses (Capatina et al., 2021; Boiangiu et al., 2022). All test compounds were dissolved in 1% dimethyl sulfoxide (DMSO) and administered *via* immersion for 1 h once daily over eight consecutive days. Control animals were exposed to unchlorinated water containing 1% DMSO alone.

### Ethic statement

All experimental procedures were conducted in accordance with EU Directive 2010/63/EU and were approved by the Ethics Committee on Animal Research of the Alexandru Ioan Cuza University of Iasi, Faculty of Biology (Approval No. BIO-UAIC-1714/6 July 2023). Animal health and welfare were closely monitored throughout the study, and no mortality or signs of pain or distress were observed during the experimental period. The study was designed and reported in compliance with the ARRIVE guidelines (Percie du Sert et al., 2020) to ensure transparency and reproducibility in animal research.

### Behavioral assays

Zebrafish swimming behavior during *in vivo* behavioral assays was recorded using a Logitech C922 Pro HD Stream camera (Logitech, Lausanne, Switzerland). Behavioral parameters were analyzed using ANY-maze behavioral tracking software, version 7.48 (Stoelting Co., Wood Dale, IL, United States).

### Novel tank diving test (NTT)

The novel tank diving test (NTT), as described by Cachat et al. (2010) is a widely used behavioral paradigm for assessing anxiety-like behavior and locomotor activity in zebrafish. In the present study, the test was performed using a 1.5 L trapezoidal tank (15.2 × 27.9 × 7.1 cm), which was virtually divided into top and bottom zones by a horizontal reference line. Individual zebrafish were gently introduced into the tank, and their behavior was recorded for 6 min. The primary behavioral endpoints included time spent in the top zone (s), the top/bottom time ratio, total distance traveled (m), and mean swimming velocity (m/s).

### Y-maze test

The Y-maze test was employed to assess novelty-seeking behavior, spatial exploration, and locomotor activity, as previously described by Boiangiu et al. (2022). Experiments were conducted in a Y-shaped glass tank (3 L) consisting of three arms (25 × 8 × 15 cm): a start arm (always open), a familiar arm (permanently open), and a novel arm, which was opened only during the test session. Each zebrafish was individually introduced into the start arm, while the novel arm remained closed during the training session (5 min). After a 1 h inter-trial interval, a test session (5 min) was conducted, during which the novel arm was opened. Behavioral endpoints included the time spent in the novel arm (expressed as a percentage of the total time spent across all arms), which served as an index of response to novelty; spontaneous alternation (%) as a measure of short-term spatial memory; and total distance traveled (m) and turn angle (°) as indicators of locomotor activity.

### Novel object recognition test (NOR)

Recognition memory in zebrafish was assessed using the novel object recognition (NOR) test, following the protocol described by Capatina et al. (2021). During the habituation phase, zebrafish were acclimated to an empty glass tank (30 × 30 × 30 cm) for 5 min per day over three consecutive days. On the fourth day (training phase), each fish was exposed to two identical objects (familiar objects, FO) placed in the tank for 10 min. After a 1 h retention interval, a test phase was conducted in which one of the familiar objects was randomly replaced with a novel object (NO). Exploratory behavior was recorded for 10 min. Recognition memory was quantified using the preference index, calculated as follows: Preference (%) = [Time exploring NO/(Time exploring FO + Time exploring NO)] × 100.

### Data analysis

All data are expressed as mean ± standard error of the mean (SEM). Statistical analyses were performed using GraphPad Prism software (version 9.0; GraphPad Software, Inc., San Diego, CA, United States). Data normality was assessed using the Shapiro-Wilk test. For multiple group comparisons, one-way ANOVA was followed by Tukey’s *post hoc* test, which controls for multiple comparisons within each dataset. Behavioral endpoints were analyzed independently, as they represent distinct functional domains. A *p*-value <0.05 was considered statistically significant. Statistical results are reported with exact *p*-values where possible, along with corresponding test statistics and degrees of freedom to ensure transparency and reproducibility.

## Results

**FIGURE 1:**
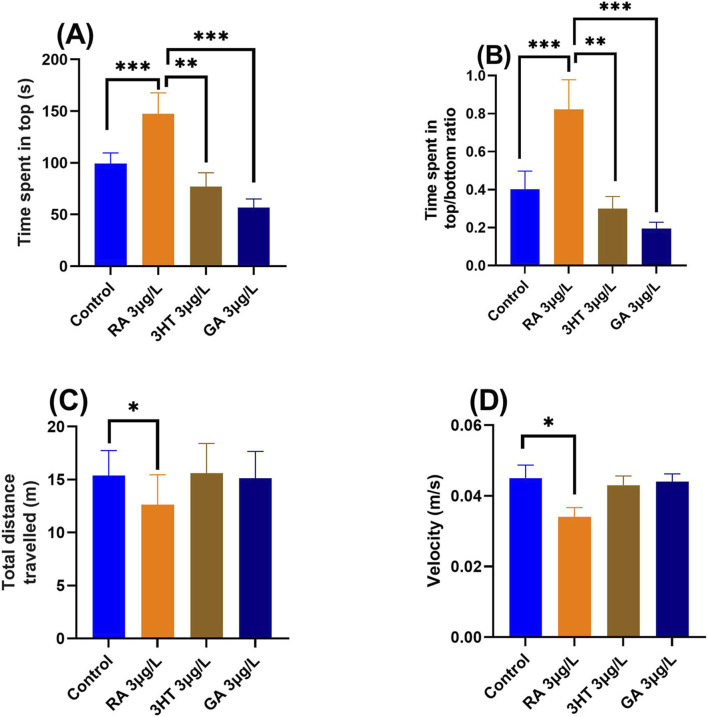
Effects of rosmarinic acid (RA, 3 μg/L), 3-hydroxytyrosol (3-HT, 3 μg/L), and gallic acid (GA, 3 μg/L) on anxiety-like behavior and locomotor activity in adult zebrafish, as assessed by the novel tank diving test (NTT). **(A,B)** RA treatment significantly reduced anxiety-like behavior compared with the control group, as indicated by increased time spent in the top zone (*p* < 0.0001) and an elevated top/bottom time ratio (p < 0.0001). **(C,D)** Locomotor activity parameters, including total distance traveled and swimming velocity, were also affected by treatment. Data are presented as mean ± SEM (n = 10 *per* group). Statistical analysis was performed using one-way ANOVA followed by Tukey’s *post hoc* test: *p < 0.01, **p < 0.001, and ***p < 0.0001. Behavioral endpoints: anxiety-like behavior-time spent in the top zone (s), top/bottom time ratio; locomotor activity-total distance traveled (m), swimming velocity (m/s). Abbreviations: RA, rosmarinic acid; 3-HT, 3-hydroxytyrosol; GA, gallic acid.

The effects of phenolic compound treatment (RA, 3-HT, and GA) on anxiety-like behavior in zebrafish were evaluated using the novel tank diving test (NTT). The analyzed behavioral parameters included time spent in the top zone, top/bottom time ratio, total distance traveled, and swimming velocity. The main effects of treatment on zebrafish behavior are illustrated in Figure 1. One-way analysis of variance (ANOVA) revealed significant treatment effects on time spent in the top zone [F (3,36) = 8.02, *p* < 0.0001; Figure 1A], top/bottom time ratio [F (3,36) = 7.85, *p* < 0.0001; Figure 1B], total distance traveled [F (3,36) = 2.78, *p* < 0.01; Figure 1C], and swimming velocity [F (3,36) = 3.14, *p* < 0.01; Figure 1D], indicating a significant overall effect of treatment across all measured parameters. Post hoc analysis showed that RA-treated zebrafish spent significantly more time in the top zone and exhibited an increased top/bottom time ratio compared with controls (both *p* < 0.0001), reflecting a pronounced anxiolytic-like behavioral profile (Figures 1A,B). In addition, RA treatment resulted in a significant reduction in locomotor activity, as evidenced by decreased total distance traveled (*p* < 0.05) and reduced swimming velocity (*p* < 0.01) (Figures 1C,D). In contrast, treatment with 3-HT and GA induced anxiety-like behavior, characterized by a significant decrease in time spent in the top zone (3-HT: p < 0.01; GA: p < 0.0001) and reduced top/bottom time ratios (3-HT: *p* < 0.01; GA: *p* < 0.0001) relative to the control group (Figures 1A,B). Notably, neither compound significantly affected total distance traveled or swimming velocity (*p* > 0.05 for both parameters), indicating that the observed anxiogenic-like responses were not confounded by alterations in general locomotor activity (Figures 1C,D). Although interaction effects between anxiety-related and locomotor parameters were not formally examined within the one-way ANOVA framework, the observed behavioral pattern suggests a dissociation between anxiety-like responses and locomotor changes, particularly in the 3-HT- and GA-treated groups.

**FIGURE 2:**
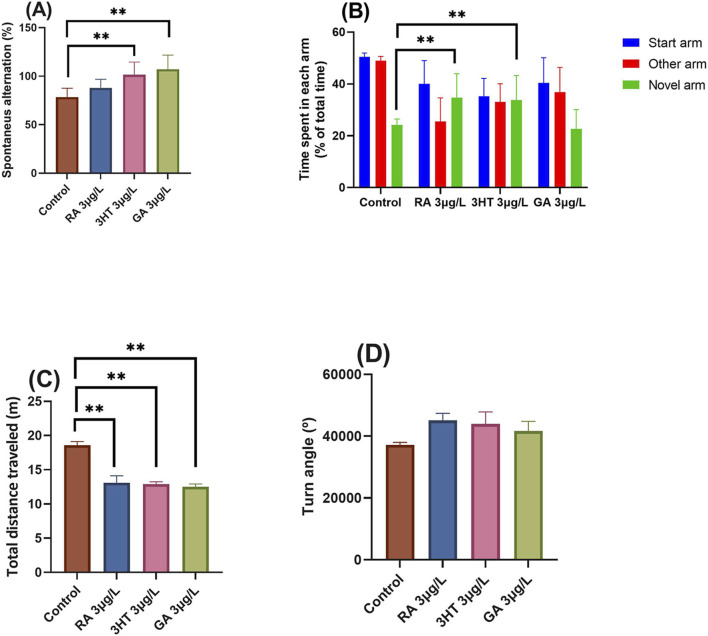
Effects of rosmarinic acid (RA, 3 μg/L), 3-hydroxytyrosol (3-HT, 3 μg/L), and gallic acid (GA, 3 μg/L) on spatial memory and novelty-driven exploration in adult zebrafish, as assessed by the Y-maze test. **(A)** Spontaneous alternation (%) as an index of spatial working memory. **(B)** Time spent in each arm, expressed as a percentage of total arm exploration time, with emphasis on the novel arm. **(C)** Total distance traveled (m) as an indicator of locomotor activity. **(D)** Turn angle (°) reflecting swimming orientation and activity patterns. Data are presented as mean ± SEM (n = 10 *per* group). Statistical analysis was performed using one-way ANOVA followed by Tukey’s *post hoc* test: **p < 0.001. Significant differences *versus* control are indicated where *p* < 0.001. Abbreviations: RA, rosmarinic acid; 3-HT, 3-hydroxytyrosol; GA, gallic acid.

To evaluate the effects of RA, 3-HT, and GA on spatial memory and novelty response, zebrafish were tested in the Y-maze paradigm. The following behavioral parameters were recorded: spontaneous alternation (%), time spent in each arm (as % of total time), total distance traveled (m) and turn angle (°). As shown in Figure 2, one-way ANOVA revealed significant overall effects of treatment on spontaneous alternation [F (3, 36) = 3.22, *p* < 0.01] (Figure 2A), time spent in each arm [F (3, 36) = 2.85, *p* < 0.01] (Figure 2B), total distance traveled [F (3, 36) = 19.48, *p* < 0.0001] (Figure 2C), and turn angle [F (3, 36) = 1.59, *p* < 0.001] (Figure 2D). Post hoc comparisons showed that 3-HT and GA significantly increased spontaneous alternation (*p* < 0.001), indicating enhanced spatial working memory (Figure 2A). Additionally, both RA and 3-HT significantly increased the time spent in the novel arm of the maze (*p* < 0.001), suggesting improved responsiveness to novelty (Figure 2B). Despite a significant reduction in total distance traveled following RA, 3-HT, and GA treatment (*p* < 0.001), this decrease in locomotion was accompanied by increased exploratory behavior in the novel arm, further supporting a memory-enhancing effect (Figure 2C). Turn angle was not significantly altered across treatment groups (Figure 2D), indicating no major disruption to basic swimming patterns. These findings collectively suggest that RA, 3-HT, and GA positively modulate spatial memory and novelty-driven exploration, with minimal impact on motor coordination.

**FIGURE 3:**
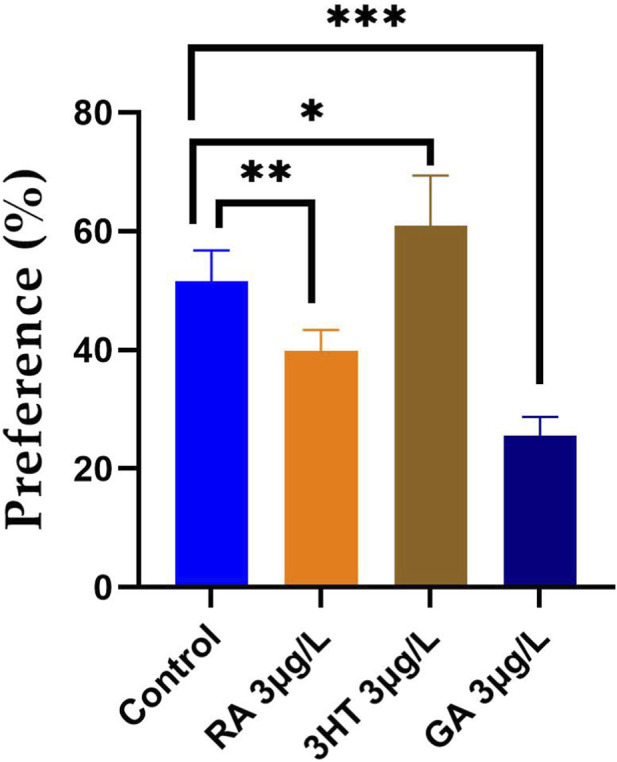
Effects of rosmarinic acid (RA, 3 μg/L), 3-hydroxytyrosol (3-HT, 3 μg/L), and gallic acid (GA, 3 μg/L) on recognition memory in adult zebrafish, as assessed by the novel object recognition (NOR) test. Novel object preference (%) was used as an index of recognition memory. Data are presented as mean ± SEM (n = 10 *per* group). Statistical analysis was performed using one-way ANOVA followed by Tukey’s *post hoc* test: *p < 0.01, **p < 0.001, and ***p < 0.0001. Abbreviations: RA, rosmarinic acid; 3-HT, 3-hydroxytyrosol; GA, gallic acid.

Recognition memory in zebrafish was assessed using the novel object recognition (NOR) test by evaluating the ability to discriminate between a familiar object and a novel object. The effects of RA, 3-HT, and GA on novel object preference are presented in Figure 3. One-way analysis of variance (ANOVA) revealed a significant overall effect of treatment on novel object preference [F (3,36) = 7.71, *p* < 0.0001; Figure 3]. Post hoc analysis showed that zebrafish treated with 3-HT displayed a significantly higher preference for the novel object compared with the control group (*p* < 0.01), indicating enhanced recognition memory. In contrast, treatment with RA and GA significantly reduced novel object preference relative to controls (RA: *p* < 0.001; GA: *p* < 0.0001), suggesting an attenuation of novelty recognition behavior. Collectively, these findings indicate that, among the phenolic compounds tested, 3-HT selectively facilitates recognition memory in adult zebrafish, whereas RA and GA do not exhibit memory-enhancing effects in this paradigm.

## Discussion

This study provides the first comprehensive *in vivo* evaluation of the anxiolytic and cognitive-modulating effects of RA, GA, and 3-HT in adult zebrafish. By extending previous *in vitro* and mammalian findings, the present data offer new insights into the neurobehavioral and pharmacological profiles of these plant-derived phenolics within a vertebrate model increasingly employed in ethnopharmacological and neurobehavioral research. Among the tested compounds, RA exerted a pronounced anxiolytic-like effect in the NTT, a well-validated assay of anxiety-related behavior in zebrafish. RA significantly increased time spent in the top zone and the top/bottom time ratio (*p* < 0.0001), classical indicators of reduced anxiety and enhanced environmental exploration. These effects were accompanied by decreased locomotor activity, reflected by reduced total distance traveled and swimming velocity. Although RA significantly increased time spent in the top zone and the top/bottom ratio, indicative of anxiolytic-like behavior, this effect was accompanied by reduced locomotor activity. This raises the possibility that the observed behavioral changes may be partially influenced by a sedative-like effect. However, the increased exploration of the top zone and enhanced novelty response in the Y-maze suggest that RA does not induce a generalized suppression of behavior. Instead, these findings may reflect a combined anxiolytic-like and mild locomotor-reducing profile. Nevertheless, further studies incorporating dose-response analyses and additional behavioral or neurochemical endpoints are warranted to clearly distinguish between anxiolytic and sedative effects. This behavioral profile is consistent with extensive evidence from rodent studies indicating that RA exerts anxiolytic and antidepressant-like effects through modulation of GABAergic and serotonergic neurotransmission, as well as through anti-inflammatory mechanisms. Suppression of pro-inflammatory cytokines and regulation of microglial activity by RA may attenuate neuroinflammation-associated anxiety, a pathway increasingly implicated in neuropsychiatric disorders. These findings are in line with prior reports demonstrating RA’s neuroprotective and antioxidant effects in zebrafish embryos and mammalian models of neurotoxicity (Zhao et al., 2020). In contrast, GA and 3-HT elicited anxiety-like responses in the NTT, evidenced by reduced time spent in the top zone and lower top/bottom ratios, without significant alterations in locomotor activity. This unexpected anxiogenic-like profile may reflect several mechanisms. One possibility is a dose-dependent hormetic response, whereby phytochemicals exert beneficial effects at low concentrations but induce stress-related responses at higher doses. Such biphasic effects have been described for several polyphenols, including resveratrol and curcumin (Calabrese, 2004; Barreiro-Sisto et al., 2024; Mattson and Leak, 2024). Alternatively, interspecies differences in pharmacodynamics may contribute to these findings. Although zebrafish share conserved neurotransmitter systems with mammals, differences in receptor distribution, enzymatic metabolism, and blood-brain barrier permeability may influence compound bioavailability and behavioral outcomes. Zebrafish-specific cytochrome P450 isoforms could also affect compound metabolism, thereby altering central nervous system exposure (Liu et al., 2023; Murari et al., 2024). Future studies incorporating dose-response analyses, chronic exposure paradigms, and neurochemical readouts such as cortisol or neurotransmitter levels would be valuable to clarify these effects. Despite their anxiogenic-like profile in the NTT, GA, and 3-HT significantly enhanced spatial working memory in the Y-maze, as evidenced by increased spontaneous alternation behavior (*p* < 0.001). These findings align with previous literature highlighting the antioxidant, anti-inflammatory, and cholinergic-modulatory properties of both compounds, which are known to support synaptic plasticity and cognitive performance. GA has demonstrated neuroprotective and anti-amyloidogenic effects in rodent models of AD (Mori et al., 2020; Alikhanzade et al., 2025), while 3-HT has shown robust antioxidant and neuroprotective activity across multiple neuronal injury models (Zhang et al., 2025). Additionally, RA and 3-HT increased time spent in the novel arm of the Y-maze, suggesting enhanced responsiveness to spatial novelty. Notably, these cognitive effects occurred alongside reduced locomotion, supporting the notion that improved performance was not driven by hyperactivity but rather by genuine enhancements in memory-related processing. Results from the NOR test further differentiated the cognitive profiles of the tested phenolics. Treatment with 3-HT significantly increased novel object preference (*p* < 0.01), indicating enhanced recognition memory and reinforcing its broad-spectrum cognitive-enhancing potential. In contrast, RA and GA reduced novel object preference relative to controls. This apparent divergence, particularly for GA, which improved Y-maze performance, may reflect task-specific cognitive modulation. The NOR test assesses object recognition, a domain potentially influenced by anxiety-related behavior and motivational state. RA’s pronounced anxiolytic effect may reduce novelty-seeking behavior, while GA’s anxiogenic-like response could promote avoidance of unfamiliar stimuli. These findings underscore the importance of employing multiple behavioral paradigms to comprehensively evaluate distinct cognitive domains and highlight the complexity of interpreting cognitive outcomes in the context of emotional state. At the molecular level, our previous *in vitro* studies demonstrated that RA, GA, and 3-HT inhibit key AD-related enzymes, including BACE1, AChE, and BChE, while modulating the expression of genes such as *PSEN*, *APOE*, and *CLU* (Ucar Akyurek et al., 2024). These molecular targets play critical roles in amyloid-β production, cholinergic neurotransmission, and amyloid clearance, providing a plausible mechanistic framework for the behavioral effects observed *in vivo*. Inhibition of BACE1 may reduce amyloidogenic processing, while cholinesterase inhibition enhances synaptic acetylcholine levels, a key determinant of memory function. Regulation of *PSEN*, *APOE*, and *CLU* may further influence amyloid aggregation and neuroinflammatory processes, supporting the translational relevance of these phenolics. However, it is important to emphasize that these mechanisms were not directly investigated here and are based on previous *in vitro* and *in silico* findings. Therefore, the proposed pathways should be considered putative, and further studies incorporating biochemical and molecular analyses are required to confirm their involvement in the zebrafish model. Collectively, this study represents the first behavioral characterization of RA, GA, and 3-HT in adult zebrafish using the NTT, Y-maze, and NOR tests. The zebrafish model, owing to its genetic tractability, translational relevance, and suitability for high-throughput screening, provides a robust platform for early-stage neuropharmacological evaluation. Nonetheless, interspecies differences necessitate follow-up studies in mammalian models to validate these findings and further define the therapeutic potential of these plant-derived phenolic compounds. This study provides novel *in vivo* evidence for distinct neurobehavioral effects of RA, GA, and 3-HT in adult zebrafish. RA exhibited robust anxiolytic activity, whereas GA and 3-HT primarily enhanced cognitive performance, particularly in spatial and recognition memory tasks. Notably, GA and 3-HT also induced anxiogenic-like responses in the novel tank diving test, highlighting compound-specific and context-dependent behavioral profiles.

Collectively, these findings underscore the multifaceted neuropharmacological potential of plant-derived phenolic compounds, e.g., RA, GA, and 3-HT, and reinforce the value of zebrafish as a sensitive and efficient model for behavioral and cognitive screening using the NTT, Y-maze, and NOR tests. The results reveal distinct neurobehavioral profiles: RA increased top-zone exploration but also reduced locomotor activity, consistent with an anxiolytic-like effect that may involve a sedative component; GA and 3-HT enhanced spatial and recognition memory despite eliciting anxiogenic-like responses in the NTT. Given the single-dose design and absence of direct mechanistic data in a zebrafish model, the findings should be considered preliminary. Further studies incorporating dose-response analyses, neurochemical measurements, and validation in mammalian models are needed to clarify the underlying mechanisms and translational relevance of these plant-derived phenolics.

## Data Availability

The original contributions presented in the study are included in the article/supplementary material, further inquiries can be directed to the corresponding author.
